# DNA Methylation Profiles of the DRD2 and NR3C1 Genes in Patients with Recent-Onset Psychosis

**DOI:** 10.1155/2022/2172564

**Published:** 2022-08-03

**Authors:** Yan Hong Piao, Yin Cui, Ling Li, Maryam Karamikheirabad, Sung-Wan Kim, Bong Ju Lee, Jung Jin Kim, Je-Chun Yu, Kyu Young Lee, Seung-Hee Won, Seung-Hwan Lee, Seung-Hyun Kim, Shi Hyun Kang, Euitae Kim, Nam-In Kang, Fatima Zahra Rami, Young-Chul Chung

**Affiliations:** ^1^Department of Psychiatry, Jeonbuk National University Medical School, Jeonju, Republic of Korea; ^2^Research Institute of Clinical Medicine of Jeonbuk National University-Biomedical Research Institute of Jeonbuk National University Hospital, Jeonju, Republic of Korea; ^3^Shanghai Mental Health Center, Shanghai Jiao Tong University School of Medicine, Shanghai, China; ^4^Department of Psychiatry, Chonnam National University Medical School, Gwangju, Republic of Korea; ^5^Department of Psychiatry, Inje University Haeundae Paik Hospital, Inje University College of Medicine, Busan, Republic of Korea; ^6^Department of Psychiatry, The Catholic University of Korea, Seoul St. Mary's Hospital, Seoul, Republic of Korea; ^7^Department of Psychiatry, Eulji University School of Medicine, Eulji University Hospital, Daejeon, Republic of Korea; ^8^Department of Psychiatry, Nowon Eulji Medical Center, Eulji University, Seoul, Republic of Korea; ^9^Department of Psychiatry, Kyungpook National University School of Medicine, Daegu, Republic of Korea; ^10^Department of Psychiatry, Inje University Ilsan Paik Hospital, Goyang, Republic of Korea; ^11^Department of Psychiatry, Korea University College of Medicine, Guro Hospital, Seoul, Republic of Korea; ^12^Department of Social Psychiatry and Rehabilitation, National Center for Mental Health, Seoul, Republic of Korea; ^13^Department of Psychiatry, Seoul National University Bundang Hospital, Seongnam, Republic of Korea; ^14^Department of Psychiatry, Maeumsarang Hospital, Wanju, Jeollabuk-do, Republic of Korea

## Abstract

**Objectives:**

Dopamine receptor D2 gene (DRD2) and glucocorticoid receptor gene (NR3C1) are implicated in the development of psychosis. We investigated methylation levels of DRD2 and NR3C1 in peripheral blood of patients with recent-onset (RO) psychosis using bisulfite pyrosequencing as well as its association with childhood trauma and rumination.

**Methods:**

In all, 51 individuals with RO psychosis and 47 healthy controls were recruited. DNA methylation levels in the targeted regions of two genes were analyzed and compared. Childhood trauma and rumination were evaluated using the Early Trauma Inventory Self-Report Short Form (ETI-SF) and Brooding Scale (BS), respectively. Correlations between the scores of the ETI-SF and BS and methylation levels were explored.

**Results:**

For DRD2, we found no significant differences between groups in terms of methylation level or association with childhood trauma or rumination. For NR3C1, we found a trend level significance for average value of all CpG sites and significant hypermethylation or hypomethylation at specific sites. There was also a significant positive correlation between the methylation level at the CpG8 site of NR3C1 exon 1F and negative symptom subscale score of the PANSS (PANSS-N).

**Conclusion:**

Epigenetic alterations of NR3C1 are associated with the pathophysiology of psychosis. Further epigenetic studies will elucidate the molecular mechanisms underpinning the pathophysiology of psychosis.

## 1. Introduction

Schizophrenia (SZ) is a complex disorder with genetic and environmental liabilities. A recent genome-wide association study of SZ revealed 108 loci associated with the disease [1]. However, the proportion of the variance in liability explained by single nucleotide polymorphisms (SNP) is small; significant genome-wide loci explain only 3.4% of the variance in liability, and the cumulative effect of common loci expressed as a polygenic risk score is estimated to explain 7% [2]. Epigenetic studies in SZ are crucial because they can elucidate how the environment affects the genetic structure of SZ. Furthermore, altered epigenetic markers can be modified by pharmaceutical and psychosocial interventions. DNA methylation, which occurs almost exclusively at CpG dinucleotides, is the most frequently studied epigenetic mechanism. DNA methylation studies generally use changes in methylation from peripheral blood cells of SZ patients at genes such as HTR1A [3], S-COMT [4], BDNF promoter 1 [5], HTR1E and COMTD1 [6], and MB-COMT [7].

The genes of interest in our study were the dopamine receptor D2 gene (DRD2) and glucocorticoid receptor gene (NR3C1). Although there is a strong association between the roles of DRD2 and NR3C1 and the development of SZ, only a few reports about DNA methylation in these genes using peripheral leukocytes have been published. Studies on DNA methylation of DRD2 have reported mixed results, including no significant difference [8] or lower methylation rates [9–11] compared to healthy controls. For NR3C1, results have varied depending on which CpG sites or components were analyzed, e.g., hypomethylation at 1D-CpG8 and hypermethylation at 1B-CpG15 and 1F-CpG21 [12] and hypomethylation or hypermethylation at component 2, including 5 CpG sites, depending on the stage of psychosis [13]. Previous studies have been limited because subjects were mainly chronic SZ patients and clinical correlates were not investigated adequately.

We investigated the association between DRD2 and NR3C1 methylation rates and childhood trauma and rumination. There is a clear link between SZ and exposure to childhood trauma [14]. Childhood trauma leads to hypothalamic–pituitary–adrenal (HPA) hyperreactivity via methylation of the NR3C1 gene, which represses NR3C1 transcription [15, 16]. Rumination, a repetitive and negatively balanced mode of thinking, has been implicated in the development of depression [17], negative [18] and positive symptoms [19], and suicidality [20] in SZ. A close correlation between childhood trauma and rumination has also been reported [21]. We hypothesized that there might be altered methylation of NR3C1 in psychosis, which in turn is associated with childhood trauma and rumination. The present study examined methylation rates of DRD2 and NR3C1 in patients with recent-onset (RO) psychosis using bisulfite pyrosequencing as well as its association with childhood trauma and rumination.

## 2. Methods

### 2.1. Participants

To be considered for inclusion, subjects were required to be between 19 and 59 years of age and meet the Diagnostic and Statistical Manual of Mental Disorders, Fifth Edition (DSM-5) [22] criteria for SZ spectrum and other psychotic disorders (SZ, schizophreniform disorder, other specified SZ spectrum and other psychotic disorders (OSSO), brief psychotic disorder, and delusional disorder). RO was defined as duration of illness ≤ 2 years. The exclusion criteria were intelligence quotient < 70, history of head trauma, serious neurological disorder (epilepsy, stroke, Parkinson's disease, and/or dementia), and significant medical illness. Participants were recruited from the Korean Early Psychosis Cohort Study (KEPS), a nationwide, multicenter, prospective, and naturalistic observational study. Age- and sex-matched healthy individuals were recruited for the control group via advertisements. All participants provided written informed consent in accordance with a protocol approved by the ethics committee of the Jeonbuk National University Hospital (approval number CUH 2014–11–002).

### 2.2. Clinical Assessment

The severity of symptoms was evaluated within a week of blood sampling using the Positive and Negative Syndrome Scale (PANSS) [23, 24] and Calgary Depression Scale for Schizophrenia (CDSS) [25, 26]. The self-rating scales employed were the Brooding Scale (BS) [27] and Early Trauma Inventory Self-Report Short Form (ETI-SF) [28]. Data for factors related to lifestyle were obtained using the Fagerstrom Test for Nicotine Dependence (FTND) [29], Alcohol Use Disorders Identification Test (AUDIT) [30], Dietary Habits Questionnaire (DHQ) [31], and Physical Activity Rating (PA-R) [32]. For simplicity, we used only the fourth item of the FTND (0, nonsmoker; 1, ≤10 cigarettes/day; 2, 11–20 cigarettes/day; 3, 21–30 cigarettes/day; and 4, ≥31 cigarettes/day) and the mean of items 1 and 2 from the AUDI, i.e., (sum of the scores on items 1 and 2)/2. The DHQ is a 20-item, self-administered questionnaire consisting of three subcategories: five items for diet regularity, six items for a balanced diet, and nine items for an unhealthy diet and eating habits. This scale was developed based on dietary guidance published by the Korean Ministry for Health, Welfare, and Family Affairs (2010). Each item is scored on a three-point scale (1, 3, and 5 points) according to the frequency of the dietary habit. Higher scores indicate better dietary habits in the respective categories. The PA-R is a questionnaire for rating a person's level of physical activity, with scores ranging from 0 (avoids walking or exercise) to 7 (runs more than 10 miles per week or spends more than 3 h per week in comparable physical activity).

### 2.3. DNA Extraction and Bisulfite Modification

Genomic DNA was extracted from the collected peripheral blood mononuclear cell samples using a QIAamp DNA Mini Kit (QIAGEN, Hilden, Germany) according to the manufacturer's instructions. All prepared samples were bisulfite-converted according to the EZ DNA Methylation Kit protocols (Zymo Research, Irvine, CA, USA).

### 2.4. Bisulfite Pyrosequencing

**Figure 1:**
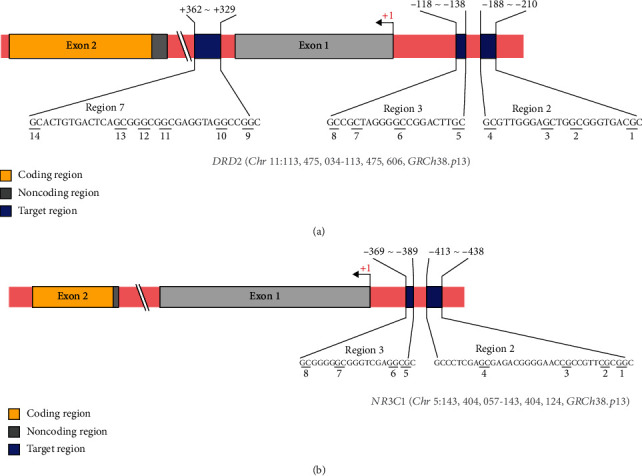
Schematic diagram showing the target locations of (a) DRD2 and (b) NR3C1.

DNA methylation was measured by pyrosequencing the polymerase chain reaction (PCR) products. Primers were designed against the putative promoter and first intron regions of DRD2, which were located from –50,955 bp to –50,382 bp of the translation start site (TSS) (chr11: 113,475,606–113,475,034) (Figure 1). A primer was designed against the exon 1_F_ region of NR3C1, which was located from –3,285 bp to –3,217 bp of the TSS (chr5: 143,404,124–143,404,057) [33] (Figure 1). Several 800–900 bp regions were initially designed using PyroMark Assay Design 2.0 software (QIAGEN). Then, key regions, i.e., those that had more transcription factor binding sites, were selected using the JASPAR database version 8 (http://jaspar.genereg.net/) [34]. Details about the PCR primers and sequencing primer are shown in Table S1. Next, 40 ng bisulfite-treated DNA was amplified in a 25 *μ*L reaction volume using the GeneAmp PCR System 9700 (Applied Biosystems, Foster City, CA, USA). Either the forward or reverse primer was biotinylated to convert the PCR product into single-stranded DNA templates, and then a sequencing primer that annealed to the single-stranded DNA template was added [35]. The PCR amplification step consisted of 45 cycles of 94°C for 30 s, 56°C for 30 s, and 72°C for 30 s; the primer sets, locations, and PCR conditions for each region are presented in Table S1. The pyrosequencing reactions were performed in a PyroMark Q48 Autoprep system (QIAGEN), and quantification of the CpG methylation (percentage of the relative light unit [RLU] of the C peak [methylated cytosine]/RLU of C peak + T peak [unmethylated cytosine]) was performed with PyroMark Q48 Autoprep 2.4.2 software (QIAGEN). When the peak value of a base exceeded 20 RLU, the pyrosequencing results were considered reliable.

### 2.5. Statistical Analysis

The assumption of normality for demographic and clinical characteristics was checked using the Shapiro–Wilk test. Because the data were not normally distributed, comparisons of demographic and clinical variables between the two groups were performed using the Mann–Whitney *U* test. Given the associations between educational attainment and DNA methylation [36], methylation rates between the two groups were compared using rank analyses of covariance with education as the covariate. Correlations between methylation rate and clinical parameters were explored using partial Spearman's rank correlation with age, sex, or chlorpromazine (CPZ) equivalent dose as covariates. Separate analyses were performed to evaluate the relationships between methylation rates and age or CPZ equivalent dose. In addition, subgroup analyses with antipsychotic-naïve plus antipsychotic-free patients, schizophrenia spectrum disorder (SSD consisting of SZ and schizophreniform disorder), or OSSO were conducted. In subgroup analyses, methylation rates between the two groups were compared using rank analyses of covariance with age and education as covariate. Comparison of methylation rates between the gender was also performed. Statistical significance was defined at the 95% level (*p* < 0.05). False discovery rate (FDR) corrections were used to limit type I error due to multiple comparisons.

## 3. Results

Demographic and clinical characteristics of the participants (51 patients with RO psychosis and 47 controls) are described in Table 1. Diagnoses included SZ (29.51%), schizophreniform disorder (31.15%), OSSO (22.95%), brief psychotic disorder (9.84%), and delusional disorder (6.56%). The education levels of the two groups differed significantly.

**Figure 2:**
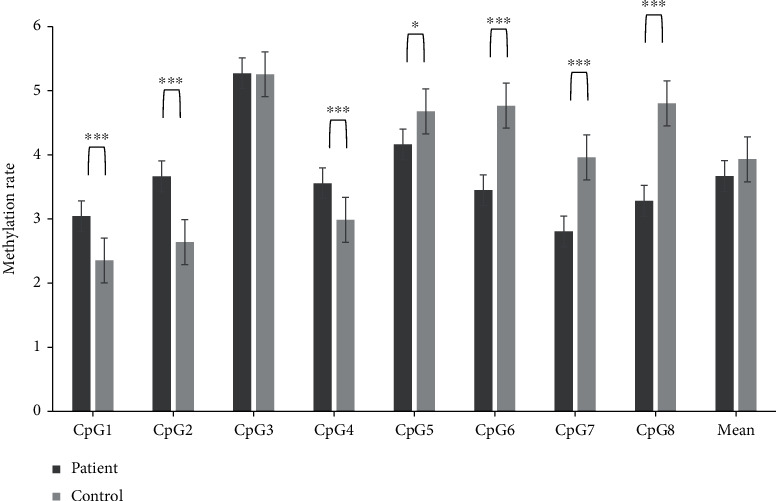
Comparison of methylation rate of NR3C1 between patients and controls.

The methylation rates of DRD2 are shown in Table 2. We found significant differences in DRD2 methylation between the two groups at CpG5 (*p* = 0.01) and CpG6 (*p* = 0.045) based on uncorrected *p* values. However, there were no significant CpG sites after FDR correction (Table 2). The results were the same as with subgroup analyses, i.e., SSD vs. controls and OSSO vs. controls (Table S2-3). We also found significant (*p* ≤ 0.001 unless otherwise noted) differences (uncorrected *p* value) for NR3C1 methylation rate between the two groups at CpG1 (*p* = 0.014), CpG2, CpG4, CpG5 (*p* = 0.018), CpG6, CpG7, and CpG8. The average values of all CpG sites showed trend level significance between the patient and control groups. The average values were less than 5% regardless of diagnosis. The results were the same after FDR correction (Figure 2 and Table S4). In the subgroup analysis, results with SSD vs. controls were exactly the same (Table S5). However, results with OSSO vs. controls were different in that significant differences were found only at CpG1, CpG2, and CpG4 (Table S6). In gender subgroup analyses, we found no significant FDR corrected *p* values for both genes in patient and control groups (Table S7-10).

**Figure 3:**
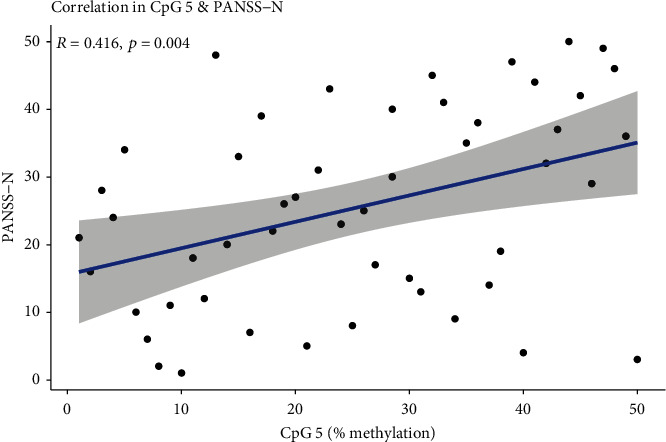
Correlation between methylation rate of NR3C1 and PANSS-N in patients.

We found no significant partial correlations (FDR corrected) between DRD2 methylation rate and clinical parameters in the patient group adjusted for age, sex, and CPZ equivalent. However, at the uncorrected *p* value, there were significant correlations of several CpG sites with the emotional abuse subscale score of the ETI-SF (ETI-SF-E), BS, and positive symptom subscale score of the PANSS (PANSS-P) (Table S11). For NR3C1, significant positive correlation was found only between the CpG5 and negative symptom subscale score of the PANSS (PANSS-N) (*r* = 0.416, FDR = 0.033) (Figure 3 and Table 3). Several other CpG sites were also significantly associated with the PANSS-N using the uncorrected *p* value (Table S12). Furthermore, in antipsychotic-naïve and -free patients, we found significant positive correlations between methylation rates of DRD2 and cognitive subscale and total scores of the BS (BS-C and BS-T) and ETI-SF-E in patients (Table S13).

**Figure 4:**
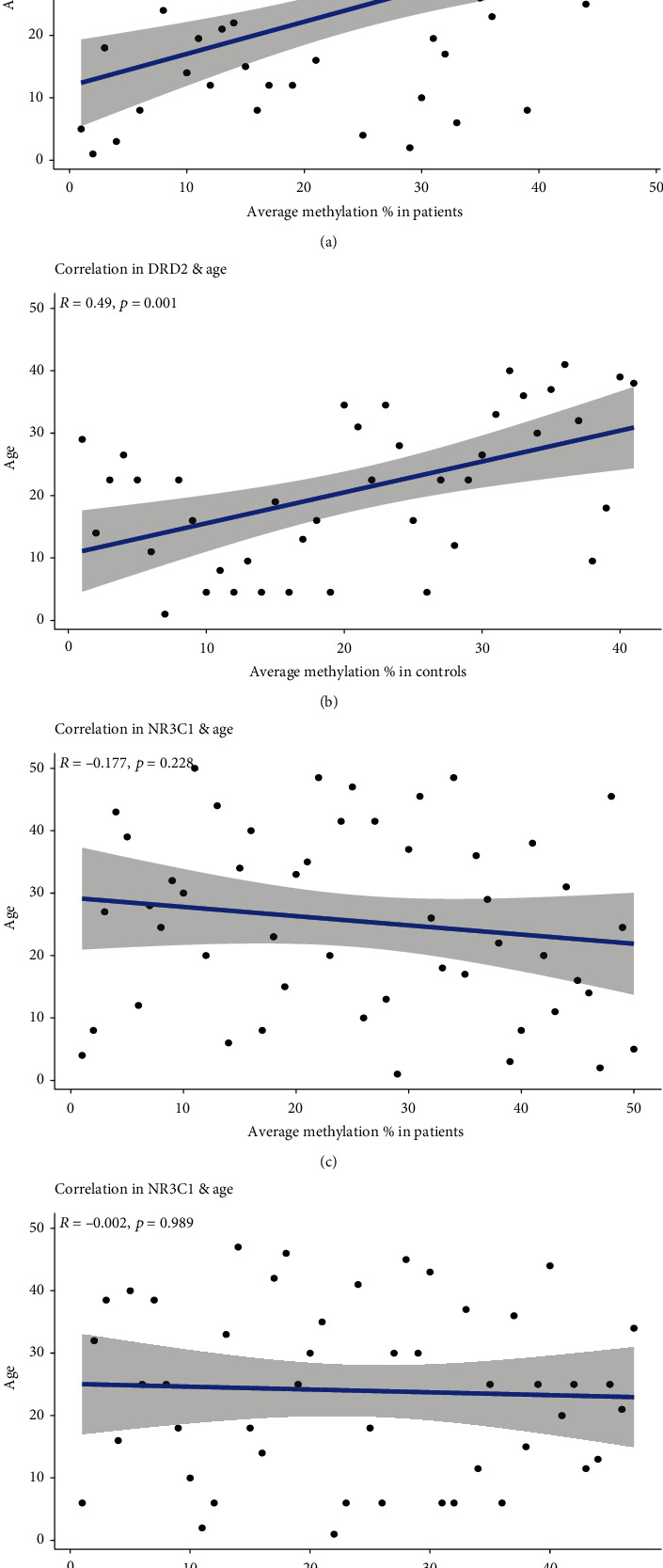
Correlations between methylation rate of DRD2 gene and (a) age in patients and (b) age in controls and between methylation rate of NR3C1 gene and (c) age in patients and (d) age in controls.

In the control group adjusted for age and sex, DRD2 methylation rates at CpG11 and CpG13 had significant negative partial correlations (FDR corrected) with BS-C (*r* = –0.432, FDR = 0.045 and *r* = –0.45, FDR = 0.045, respectively) (Table 4). Using the uncorrected *p* value, we found significant correlations for several CpG sites with the ETI-SF and BS (Table S14). For NR3C1, we found no significant correlations using FDR corrected *p* values; however, we found significant correlations between CpG4 and the ETI-SF-S and BS-C using the uncorrected *p* values (Table S15). There were significant positive correlations between the age and average DRD2 methylation rates as well as with several specific rates in both patients and controls (Figure 4 and Table S16-17). We found no significant correlations between age and NR3C1 methylation rate in patients or controls (Table S18-19) or between CPZ equivalent dose in patients and methylation rate for either DRD2 or NR3C1.

## 4. Discussion

Epigenetics may provide a promising complement to psychosis studies of DNA sequence variation. It could shed light on how environmental factors affect gene expression and elucidate the pathophysiology of psychosis. We investigated DNA methylation rates at two candidate regions (DRD2 and NR3C1) in patients with RO psychosis and healthy controls. The associations between gene methylation rates and childhood trauma and rumination were also explored.

We found no significant differences (FDR corrected) in DRD2 between groups in contrast with previous studies [9–11]. In two previous studies [9, 11], the target region of DRD2 differed (only the CpG3, CpG4, CpG5, and CpG6 sites overlapped with CpG1, CpG2, CpG3, and CpG4 used in the present study). Moreover, participants were older with chronic SZ and took higher CPZ equivalent doses than patients in the present study. Kordi-Tamandani et al. [10] used methylation-specific PCR instead of pyrosequencing and reported only the global methylation rate. Therefore, our results cannot be compared directly to those of three previous studies reporting lower methylation rate of DRD2 in patients compared to controls [9–11]. It could be speculated that methylation status of DRD2 may be different at different stages of illness in psychosis. Considering significant correlations of age with methylation rate of DRD2 in both patient and control groups, we further performed the same analysis with age being added as covariate. However, results were the same. In addition, no significant correlations with clinical parameters were reported. These results may have been caused by differences in the stages of psychosis or target regions of DRD2. Alternatively, medication may have affected the results although it was controlled as a covariate. This explanation is supported by the analyses of antipsychotic-naïve and -free patients. Finally, DRD2 methylation rate in the blood may not accurately reflect the status of methylation in the brain. Unexpectedly, we found significant negative correlations between the DRD2 methylation rates at CpG11 and CpG13 and BS-C in the control group. Because the control group had a significantly lower BS score than the patient group, the implication of this finding remains unclear.

For NR3C1, we found a trend level significance for average value of all CpG sites. Furthermore, significant hypermethylation or hypomethylation at specific sites was observed. This suggests that different CpG loci of NR3C1 may participate in psychosis through different regulatory mechanisms. We know of only two studies that measured DNA methylation of NR3C1 in SZ. One Chinese study reported no significant difference for the average value of CpG sites covering region 1_F_ between patients with SZ and controls [12]. However, patients had significantly higher DNA methylation at CpG21 than controls. Different target regions (–3,285 to –3,487 relative to TSS) than ours (–3,217 to –3,285 relative to TSS) were also used. Furthermore, patients were much older than in our study, and the severity of symptoms was not provided. A Western study found that patients with first-episode psychosis (FEP) had significantly lower methylation of component 2 than other subgroups of participants [13]. The CpG1, CpG2, CpG3, and CpG4 sites were exactly the same as those in our study, and the CpG6, CpG7, CpG8, and CpG9 sites corresponded to our CpG5, CpG6, CpG7, and CpG8 sites, respectively. In the Misiak et al. study [13], compared to healthy controls, methylation rates of CpG2 and CpG4 were significantly lower in patients with FEP but higher in acutely relapsed patients with SZ. Therefore, the result of methylation rate at CpG2 and CpG4 in acutely relapsed patient does match exactly with our result. In the subgroup analyses, results with SSD vs. controls were same, but results with OSSO vs. controls were different. These findings signify that our initial results were mainly driven by patients with SSD and methylation status of NR3C1 in OSSO is different from SSD. Considering that patients with psychotic disorder not otherwise specified, equivalent to OSSO, show different clinical characteristics and better outcome compared to SZ [37], a separate study on NR3C1 methylation in OSSO is warranted. Interestingly, we found a significant positive correlation between methylation at CpG5 and PANSS-N. This suggests that more severe negative symptoms are associated with reduced expression of glucocorticoid receptor. Methylation rates of NR3C1 exon 1_F_ region are closely related to trauma exposure, adversity, nonabuse suicide, and anxiety disorders [38–42]. Thus, stress or childhood trauma may lead to HPA hyperreactivity via methylation of NR3C1, which represses NR3C1 transcription. Unfortunately, we were unable to find an association between methylation of the exon 1_F_ region of NR3C1 and childhood trauma or rumination, although it was significant with the uncorrected *p* value. It may be that recent life stress rather than childhood trauma affected the results. Notably, two studies have reported an association between NR3C1 methylation and childhood adversity in psychosis [13, 43].

This study had several limitations. As with most previous studies, we selected a few candidate regions of two genes based on relevant findings. However, the locations of the regions do not match exactly with those of previous studies, which makes direct comparison difficult. This highlights the need to measure genome-wide methylation rates using microarray or methylation sequencing methods. In addition, our participants were heterogeneous in terms of diagnosis. The effects of medication were also not fully controlled. Although we did not find significant correlations between methylation rates of the two genes and CPZ equivalent dose, there is increasing evidence that several antipsychotic drugs, with the exception of haloperidol, display demethylating properties [44–46]. Therefore, antipsychotic-naïve or -free patients should be enrolled in future studies to avoid confounding effects. Finally, our methylation values were not adjusted for variation in the proportion of cell type. In blood, DNA methylation rate between the most frequent cell types varies from 3.5% up to 42% in data from about 450,000 preselected CpGs [47]. Adjusting cellular heterogeneity has usually been applied to data measured via genome-wide analyses with Illumina BeadChip microarrays. However, considering region-specific differential DNA methylation between white blood cell subtypes [48], adjusting for proportion of cell type seems necessary for bisulfite pyrosequencing data.

Despite these caveats, our study is the first trial to measure DNA methylation rates of two key genes in individuals with RO psychosis and explore its association with childhood trauma. In addition, we carefully controlled for various confounding factors related to lifestyle behaviors such as smoking, drinking, dietary habits, and physical activity. We identified aberrant DNA methylation rates in patients with RO psychosis compared to healthy controls at several CpG sites of the NR3C1 exon 1_F_ region but not at the target regions of DRD2. Although the functional significance of this difference in methylation is unclear, it suggests that epigenetic aberrations of NR3C1 are associated with the presence of RO psychosis and negative symptoms. Improved understanding of epigenetic liabilities would help prevent or reverse certain disease processes related to psychosis.

## Figures and Tables

**Table 1:** Demographic and clinical characteristics of patients and healthy controls.

	Patients (*n* = 48 − 51)	Controls (*n* = 47)	*p* value^a^	Mann–Whitney *U*
Age	31.5 ± 12.1	30.9 ± 8.9	0.489	1296.000
Male/female	21 (41.2)/30 (58.8)	13 (27.7)/34 (72.3)	0.164	
Education	13.9 ± 2.8	15.7 ± 1.0	<0.001^∗∗∗^	1643.500
CPZ equivalent (mg/day)	334 ± 228 (*n* = 18)			
DUP (month)	16.0 ± 40.1			
DI (month)	21.3 ± 44.8			
PANSS				
Positive	19.4 ± 6.1			
Negative	13.0 ± 6.2			
General	33.5 ± 7.8			
Total	66.0 ± 15.1			
CDSS	5.9 ± 4.8			
Brooding Scale^b^				
Emotional	9.0 ± 5.0	6.3 ± 3.9	0.003^∗∗^	749.500
Cognitive	7.9 ± 4.3	4.9 ± 3.2	<0.001^∗∗∗^	639.500
Total	17.0 ± 8.7	11.2 ± 6.6	<0.001^∗∗∗^	658.500
ETI-SF^b^				
Emotional	1.9 ± 1.8	0.5 ± 1.1	<0.001^∗∗∗^	584.500
General	1.9 ± 1.9	0.8 ± 1.1	0.001^∗∗^	732.000
Physical	1.9 ± 1.7	1.2 ± 1.4	0.029^∗^	864.500
Sexual	0.4 ± 1.0	0.3 ± 0.6	0.963	1156.500
Total	5.9 ± 5.1	2.7 ± 2.7	<0.001^∗∗∗^	653.000
SOFAS	57.7 ± 13.4			
FTND^b^	0.4 ± 0.7	0.2 ± 0.5	0.050	961.500
AUDIT	1.1 ± 1.2	1.2 ± 1.0	0.584	1274.000
DHQ	61.5 ± 12.2	56.6 ± 13.1	0.077	831.000
PA-R^c^	1.3 ± 1.3	1.8 ± 2.0	0.349	1248.500

Note: AUDIT: Alcohol Use Disorders Identification Test; PA-R: activities during the past month; BCSS: brief core schema scales; CDSS: Calgary Depression Scale for Schizophrenia; CPZ: chlorpromazine; DHQ: Dietary Habits Questionnaire; ETI-SF: Early Trauma Inventory Self-Report Short Form; FTND: Fagerstrom Test for Nicotine Dependence; PANSS: Positive and Negative Syndrome Scale; PA-R: Physical Activity Rating; SOFAS: Sociooccupational Functioning Assessment Scale. ^a^Mann–Whitney *U* test. ^b^*n* = 49. ^c^*n* = 48. ^∗^ < 0.05, ^∗∗^ < 0.01, and ^∗∗∗^ < 0.001.

**Table 2:** Comparison of methylation rate of DRD2 between patients and controls^c^.

CpG sites	Patient (*n* = 48 − 51)	Control (*n* = 41 − 47)	*F* (df)	*p* value^a^	*p* value^b^
CpG1	24 ± 3.9	22.8 ± 2.9	1.013 (1, 96)	0.317	0.494
CpG2	33.6 ± 4.4	31.8 ± 2.9	1.626 (1, 96)	0.205	0.452
CpG3	10.8 ± 2.7	9.7 ± 1.4	0.766 (1, 95)	0.384	0.494
CpG4	12.4 ± 2.8	11.2 ± 1.6	3.725 (1, 87)	0.057	0.213
CpG5	5.4 ± 1.3	5.9 ± 1.3	6.923 (1, 96)	0.010^∗^	0.149
CpG6	5.2 ± 1.1	5.3 ± 1.1	4.116 (1, 96)	0.045^∗^	0.213
CpG7	4.6 ± 1.1	4.6 ± 0.7	2.117 (1, 96)	0.149	0.447
CpG8	4.3 ± 0.9	4.4 ± 0.8	3.832 (1, 96)	0.053	0.213
CpG9	11.7 ± 3.4	10.7 ± 1.7	0.298 (1, 96)	0.586	0.628
CpG10	16.6 ± 3.2	15.9 ± 2.1	0.730 (1, 96)	0.395	0.494
CpG11	16.3 ± 3.3	15.4 ± 1.7	0.949 (1, 96)	0.332	0.494
CpG12	18.4 ± 2.0	17.6 ± 1.3	0.470 (1, 96)	0.495	0.571
CpG13	11.8 ± 3.0	11.1 ± 1.4	0.948 (1. 96)	0.333	0.494
CpG14	5.3 ± 1.5	4.9 ± 0.7	1.586 (1, 96)	0.211	0.452
Mean	12.8 ± 2.0	12.2 ± 1.02	0.179 (1, 87)	0.674	0.674

^a^Uncorrected *p* value. ^b^False discovery rate adjusted *p* value. ^c^Ranked ANCOVA: a statistic controlling for the potential confounding effects of education. ^∗^ < 0.05, ^∗∗^ < 0.01, and ^∗∗∗^ < 0.001.

**Table 3:** Correlation between methylation rate of NR3C1 and clinical parameters in patients^b^.

CpG sites	BS-E (n=48)		BS-C (n=48)		BS-T (n=48)		ETI-SF-G (n=48)		ETI-SF-P (n=48)		ETI-SF-E (n=48)		ETI-SF-S (N=48)		ETI-SF-T (N=48)		PANSS-P (n=50)		PANSS-N (n=50)		PANSS-G (n=50)		PANSS-T (n=50)	
	r	p-value^a^	r	p-value^a^	r	p-value^a^	r	p-value^a^	r	p-value^a^	r	p-value^a^	r	p-value^a^	r	p-value^a^	r	p-value^a^	r	p-value^a^	r	p-value^a^	r	p-value^a^
CpG 1	-0.033	0.907	-0.075	0.705	-0.059	0.900	-0.051	0.830	0.071	0.680	-0.070	0.831	-0.124	0.708	-0.061	0.792	0.026	0.862	-0.056	0.884	-0.072	0.934	-0.042	0.917
CpG 2	-0.137	0.782	-0.132	0.583	-0.147	0.601	0.115	0.830	-0.087	0.680	-0.223	0.738	-0.161	0.708	-0.134	0.792	0.112	0.739	-0.129	0.584	-0.108	0.934	-0.018	0.917
CpG 3	0.022	0.907	-0.137	0.583	-0.037	0.911	0.069	0.830	0.063	0.680	0.044	0.869	0.127	0.708	0.123	0.792	0.093	0.739	-0.017	0.911	-0.051	0.934	-0.031	0.917
CpG 4	-0.018	0.907	-0.010	0.948	-0.013	0.932	0.197	0.802	0.093	0.680	0.014	0.928	-0.011	0.942	0.092	0.792	0.045	0.861	0.041	0.884	-0.102	0.934	0.016	0.917
CpG 5	0.167	0.782	0.217	0.387	0.247	0.363	-0.175	0.802	0.199	0.531	0.149	0.738	0.171	0.708	0.067	0.792	-0.213	0.696	0.416^∗∗^	0.033∗	-0.021	0.934	0.084	0.917
CpG 6	0.119	0.782	0.252	0.387	0.217	0.363	-0.169	0.802	0.180	0.531	0.126	0.740	0.133	0.708	0.051	0.792	-0.198	0.696	0.301^∗^	0.090	-0.017	0.934	0.042	0.917
CpG 7	0.172	0.782	0.242	0.387	0.240	0.363	0.010	0.948	0.262	0.531	0.168	0.738	0.091	0.708	0.140	0.792	-0.172	0.696	0.309^∗^	0.090	0.096	0.934	0.092	0.917
CpG 8	0.164	0.782	0.207	0.387	0.212	0.363	-0.069	0.830	0.210	0.531	0.159	0.738	0.044	0.869	0.093	0.792	-0.152	0.696	0.352^∗^	0.069	0.012	0.934	0.088	0.917
Mean	0.059	0.907	0.106	0.628	0.104	0.743	-0.072	0.830	0.157	0.548	0.098	0.782	0.092	0.708	0.040	0.792	-0.084	0.739	0.236	0.200	0.043	0.934	0.088	0.917

Note: BS-E, -C, and –T: Brooding Scale-Emotion, -Cognition, and -Total; ETI-SF-G, -P, -E, -S, and T: Early Trauma Inventory Self-Report Short Form-General, -Physical, -Emotion, -Sexual, and -Total; PANSS-P, -N, -G, and -T: Positive and Negative Syndrome Scale-Positive, -Negative, -General, and -Total. ^a^False discovery rate adjusted *p* value. ^b^Spearman partial correlation analysis was performed with a covariate of age, gender, and CPZ. ^∗^ < 0.05, ^∗∗^ < 0.01, and ^∗∗∗^ < 0.001.

**Table 4:** Correlation between methylation rate of DRD2 and clinical parameters in controls^b^.

CpG sites	BS-E (n =41)		BS-C (n =41)		BS-T (n =41)		ETI-SF-G (n =41)		ETI-SF-P (n =41)		ETI-SF-E (n =41)		ETI-SF-S (n =41)		ETI-SF-T (N =41)	
	*r*	*p* value^a^	*r*	*p* value^a^	*r*	*p* value^a^	*r*	*p* value^a^	*r*	*p* value^a^	*r*	*p* value^a^	*r*	*p* value^a^	*r*	*p* value^a^
CpG1	0.208	0.384	0.218	0.391	0.239	0.269	0.072	0.826	0.263	0.330	0.184	0.708	0.092	0.785	0.186	0.482
CpG2	-0.032	0.877	-0.175	0.508	-0.088	0.780	0.125	0.718	0.086	0.717	-0.116	0.708	0.247	0.582	0.087	0.726
CpG3	0.391^∗^	0.209	0.169	0.508	0.333^∗^	0.114	0.375^∗^	0.279	0.350^∗^	0.216	0.382^∗^	0.245	0.071	0.834	0.441^∗∗^	0.075
CpG4	0.068	0.852	0.043	0.852	0.065	0.780	0.223	0.678	0.369^∗^	0.216	0.144	0.708	0.232	0.582	0.358^∗^	0.190
CpG5	0.172	0.493	-0.106	0.601	0.058	0.780	0.181	0.678	0.190	0.461	0.109	0.708	0.234	0.582	0.260	0.482
CpG6	0.093	0.784	0.113	0.601	0.118	0.714	0.199	0.678	0.145	0.568	0.106	0.708	0.114	0.785	0.194	0.482
CpG7	-0.160	0.496	-0.148	0.521	-0.197	0.384	0.147	0.718	0.223	0.390	0.023	0.940	-0.106	0.785	0.149	0.611
CpG8	-0.055	0.853	0.028	0.864	-0.006	0.972	0.243	0.678	0.218	0.390	0.135	0.708	0.180	0.753	0.217	0.482
CpG9	-0.250	0.384	-0.218	0.391	-0.271	0.204	-0.117	0.718	-0.045	0.785	-0.122	0.708	0.021	0.898	-0.129	0.651
CpG10	-0.323^∗^	0.334	-0.356^∗^	0.131	-0.377^∗^	0.114	-0.049	0.826	0.074	0.717	-0.155	0.708	-0.130	0.785	-0.080	0.726
CpG11	-0.295	0.340	-0.432^∗∗^	0.045	-0.367^∗^	0.114	-0.181	0.678	0.071	0.717	-0.303	0.459	0.044	0.847	-0.117	0.651
CpG12	-0.243	0.384	-0.325^∗^	0.163	-0.335^∗^	0.114	0.042	0.826	0.075	0.717	-0.115	0.708	-0.255	0.582	0.013	0.936
CpG13	-0.213	0.384	-0.450^∗∗^	0.045	-0.351^∗^	0.114	0.036	0.826	0.325^∗^	0.216	-0.013	0.940	-0.056	0.847	0.197	0.482
CpG14	-0.220	0.384	-0.303	0.183	-0.299	0.160	-0.140	0.718	0.161	0.545	0.020	0.940	-0.105	0.785	-0.017	0.936
Mean	-0.026	0.877	-0.144	0.521	-0.074	0.780	0.096	0.767	0.260	0.330	0.066	0.861	0.170	0.753	0.197	0.482

Note: BS-E, -C, and -T: Brooding Scale-Emotion, -Cognition, and -Total; ETI-SF-G, -P, -E, -S, and -T: Early Trauma Inventory Self-Report Short Form-General, -Physical, -Emotion, -Sexual, and -Total. ^a^False discovery rate adjusted *p* value. ^b^Spearman partial correlation analysis was performed with a covariate of age and gender. ^∗^ < 0.05, ^∗∗^ < 0.01, and ^∗∗∗^ < 0.001.

## Data Availability

The data used to support the findings of this study are available from the corresponding author upon request.

## Supplementary Materials

Supplementary Materials Table S1: PCR primers and sequencing primers and PCR condition. Table S2–Table S6: subgroup comparison analysis. Table S7–Table S10: gender comparison analysis. Table S11–Table S19: correlation analysis.
