# Supplementary material for: DNA Methylation Profiles of the DRD2 and NR3C1 Genes in Patients with Recent-Onset Psychosis
Source: Dis Markers. 2022 Aug 3;2022:2172564. doi: 10.1155/2022/2172564 (PMC9365600; doi:10.1155/2022/2172564)
Supplement: Supplementary Materials — Table S1: PCR primers and sequencing primers and PCR condition. Table S2–Table S6: subgroup comparison analysis. Table S7–Table S10: gender comparison analysis. Table S11–Table S19: correlation analysis. [file 2172564.f1.docx]

**Table S1. PCR primers and sequencing primers, and PCR condition.**

| **Gene** | **Region** | **Location** | **Primers** | **Sequencing primer** | **Annealing temperature (℃)** | **CpG number** |
| --- | --- | --- | --- | --- | --- | --- |
| DRD2 | 2 | 113 475 606 – 113 475 584  (-50955 to -50926 of the TSS) | F : TTTGGTTTTTGAGTTTTTAAAGGAGAAGAT  R : CCCCACCAAAAAAACTATACCT | GGGTGTGGGTGGGAG | 56 | 4 |
|  | 3 | 113 475 534 – 113 475 514  (-50883 to -50863 of the TSS) | F : AGTTTATTATTTTGGGTGTG  R : CCCCACCAAAAAAACTATACCT | GGAATTTTTTAATTTTTTTTAT | 56 | 4 |
|  | 7 | 113 475 067 – 113 475 033  (-50416 to -50382 of the TSS) | F : GGGAGTTTTATGGTTTGAAGGTAAGTA  R : ACCCCACAAACTTCTAATCCTAACCT | ATGGTTTGAAGGTAAGTAT | 56 | 6 |
| NR3C1 | 2 | 143 404 124 – 143 404 090  (-3285 to -3251 of the TSS) | F : GTTGTTATTAGTAGGGGTATTG  R : AAAACCCACCTAATCTCTCTAAAAC | ACTCCAAACCCACTC | 56 | 4 |
|  | 3 | 143] 404 075 – 143 404 057 | F: GTTGTTATTAGTAGGGGTATTG | CTTTTCTCCCTACCTCCTTCC | 56 | 4 |
|  |  | (-3236 to -3217 of the TSS) | R: AAAACCCACCTAATCTCTCTAAAAC |  |  |  |

Note: CpG, Cytosine-phosphate-guanine; DRD2, Dopamine Receptor D2; F, Forward; NR3C1, Nuclear Receptor Subfamily 3 Group C Member 1; R, Reverse; TSS, Translation Start Site.

**Table S2. Comparison of methylation rate of DRD2 between patients with SSD and controls^c^**

| **CpG sites** | **Patient (n=36-37)** | **Control (n=41-47)** | **F (df)** | **p-value^a^** | **p-value^b^** |
| --- | --- | --- | --- | --- | --- |
| CpG1 | 23.8±3.9 | 22.8±2.9 | 0.367 (1, 82) | 0.546 | 0.910 |
| CpG2 | 33.6±4.5 | 31.8±2.9 | 0.623 (1, 82) | 0.432 | 0.910 |
| CpG3 | 10.9±2.7 | 9.7±1.4 | 1.348 (1, 81) | 0.249 | 0.747 |
| CpG4 | 12.6±3.1 | 11.2±1.6 | 4.404 (1, 75) | 0.039^*^ | 0.510 |
| CpG5 | 5.5±1.2 | 5.9±1.3 | 3.433 (1, 82) | 0.068 | 0.510 |
| CpG6 | 5.3±1.0 | 5.3±1.1 | 1.979 (1, 82) | 0.163 | 0.611 |
| CpG7 | 4.7±1.1 | 4.6±0.7 | 0.786 (1, 82) | 0.378 | 0.910 |
| CpG8 | 4.3±0.9 | 4.4±0.7 | 2.718 (1, 82) | 0.103 | 0.515 |
| CpG9 | 11.9±3.5 | 10.7±1.6 | 0.006 (1, 82) | 0.937 | 0.955 |
| CpG10 | 16.8±3.2 | 15.9±2.1 | 0.049 (1, 82) | 0.825 | 0.955 |
| CpG11 | 16.6±3.5 | 15.4±1.7 | 0.029 (1, 82) | 0.865 | 0.955 |
| CpG12 | 18.4±2.2 | 17.6±1.3 | 0.082 (1, 82) | 0.775 | 0.955 |
| CpG13 | 12.0±3.2 | 11.1±1.4 | 0.090 (1, 82) | 0.765 | 0.955 |
| CpG14 | 5.4±1.6 | 4.9±0.7 | 0.437 (1, 82) | 0.511 | 0.910 |
| Mean | 13.0±2.1 | 12.2±1.0 | 0.003 (1, 75) | 0.955 | 0.955 |

^a^Uncorrected p-value; ^b^False Discovery Rate adjusted p-value; ^c^Ranked ANCOVA: A statistic controlling for the potential confounding effects of education; SSD, Schizoprenia Spectrum Disorder (schizophrenia and schizophreniform disorder)

* ＜ 0.05, ** ＜ 0.01, *** ＜ 0.001

**Table S3. Comparison of methylation rate of DRD2 between patients with OSSO and controls^c^**

| **CpG sites** | **Patient (n=12-14)** | **Control (n=41-47)** | **F (df)** | **p-value^a^** | **p-value^b^** |
| --- | --- | --- | --- | --- | --- |
| CpG1 | 24.6±3.8 | 22.8±2.9 | 2.885 (1, 59) | 0.095 | 0.238 |
| CpG2 | 33.5±4.3 | 31.8±2.9 | 3.667 (1, 59) | 0.060 | 0.180 |
| CpG3 | 10.4±2.6 | 9.7±1.4 | 0.039 (1, 59) | 0.844 | 0.844 |
| CpG4 | 11.8±1.5 | 11.2±1.6 | 0.342 (1, 51) | 0.561 | 0.601 |
| CpG5 | 5.1±1.4 | 5.9±1.3 | 8.454 (1, 59) | 0.005^**^ | 0.075 |
| CpG6 | 4.7±1.1 | 5.3±1.1 | 7.162 (1, 59) | 0.010^*^ | 0.075 |
| CpG7 | 4.3±1.0 | 4.6±0.7 | 1.948 (1, 59) | 0.168 | 0.251 |
| CpG8 | 4.3±0.9 | 4.4±0.7 | 3.695 (1, 59) | 0.059 | 0.180 |
| CpG9 | 11.0±3.0 | 10.7±1.6 | 0.816 (1, 59) | 0.370 | 0.441 |
| CpG10 | 16.2±3.3 | 15.9±2.1 | 1.806 (1, 59) | 0.184 | 0.251 |
| CpG11 | 15.3±2.8 | 15.4±1.7 | 3.761 (1, 59) | 0.057 | 0.180 |
| CpG12 | 18.4±1.6 | 17.6±1.3 | 2.147 (1, 59) | 0.148 | 0.251 |
| CpG13 | 11.4±2.7 | 11.1±1.4 | 2.564 (1, 59) | 0.115 | 0.246 |
| CpG14 | 4.9±1.2 | 4.9±0.7 | 1.987 (1, 59) | 0.164 | 0.251 |
| Mean | 12.3±1.7 | 12.2±1.0 | 0.778 (1, 51) | 0.382 | 0.246 |

^a^Uncorrected p-value; ^b^False Discovery Rate adjusted p-value; ^c^Ranked ANCOVA: A statistic controlling for the potential confounding effects of education; OSSO, Other Specified Schizophrenia spectrum and Other psychotic disorder.

* ＜ 0.05, ** ＜ 0.01, *** ＜ 0.001

**Table S4. Comparison of methylation rate of NR3C1 between patients and controls^c^**

| **CpG sites** | **Patient (n=50-51)** | **Control (n=47)** | **F (df)** | **p-value^a^** | **p-value^b^** |
| --- | --- | --- | --- | --- | --- |
| CpG1 | 3.0±1.0 | 2.4±1.0 | 18.340 (1, 95) | < 0.001^***^ | < 0.001^***^ |
| CpG2 | 3.7±1.5 | 2.6±0.7 | 45.360 (1, 95) | < 0.001^***^ | < 0.001^***^ |
| CpG3 | 5.3±0.8 | 5.3±0.5 | 0.393 (1, 95) | 0.532 | 0.532 |
| CpG4 | 3.6±0.8 | 3.0±0.6 | 21.700 (1, 96) | < 0.001^***^ | < 0.001^***^ |
| CpG5 | 4.2±0.9 | 4.7±0.7 | 6.319 (1, 96) | 0.014^*^ | 0.018^*^ |
| CpG6 | 3.5±1.4 | 4.8±1.3 | 16.775 (1, 96) | < 0.001^***^ | < 0.001^***^ |
| CpG7 | 2.8±1.3 | 4.0±1.1 | 23.507 (1, 96) | < 0.001^***^ | < 0.001^***^ |
| CpG8 | 3.3±1.7 | 4.8±1.6 | 18.074 (1, 96) | < 0.001^***^ | < 0.001^***^ |
| Mean | 3.7±0.8 | 3.9±0.6 | 3.321 (1, 95) | 0.072 | 0.080 |

^a^Uncorrected p-value

^b^False Discovery Rate adjusted p-value

^c^Ranked ANCOVA: A statistic controlling for the potential confounding effects of education.

* ＜ 0.05, ** ＜ 0.01, *** ＜ 0.001

**Table S5. Comparison of methylation rate of NR3C1 between patients with SSD and controls^c^**

| **CpG sites** | **Patient (n=36-37)** | **Control (n=47)** | **F (df)** | **p-value^a^** | **p-value^b^** |
| --- | --- | --- | --- | --- | --- |
| CpG1 | 3.1±1.1 | 2.3±1.0 | 11.711 (1, 81) | 0.001^**^ | 0.002^**^ |
| CpG2 | 3.8±1.7 | 2.6±0.7 | 34.589 (1, 81) | < 0.001^***^ | < 0.001^***^ |
| CpG3 | 5.2±0.8 | 5.2±0.5 | 0.198 (1, 81) | 0.658 | 0.658 |
| CpG4 | 3.6±0.8 | 3.0±0.6 | 15.359 (1, 82) | < 0.001^***^ | < 0.001^***^ |
| CpG5 | 4.1±0.8 | 4.7±0.7 | 7.966 (1, 82) | 0.006^**^ | 0.008^**^ |
| CpG6 | 3.4±1.2 | 4.8±1.3 | 16.733 (1, 82) | < 0.001^***^ | < 0.001^***^ |
| CpG7 | 2.7±1.2 | 4.0±1.1 | 22.718 (1, 82) | < 0.001^***^ | < 0.001^***^ |
| CpG8 | 3.1±1.5 | 4.8±1.6 | 23.529 (1, 82) | < 0.001^***^ | < 0.001^***^ |
| Mean | 3.6±0.7 | 3.9±0.6 | 4.175 (1, 81) | 0.044^*^ | 0.050 |

^a^Uncorrected p-value; ^b^False Discovery Rate adjusted p-value; ^c^Ranked ANCOVA: A statistic controlling for the potential confounding effects of education; SSD, SSD, Schizoprenia Spectrum Disorder (schizophrenia and schizophreniform disorder)

* ＜ 0.05, ** ＜ 0.01, *** ＜ 0.001

**Table S6. Comparison of methylation rate of NR3C1 between patients with OSSO and controls^c^**

| **CpG sites** | **Patient (n=14)** | **Control (n=47)** | **F (df)** | **p-value^a^** | **p-value^b^** |
| --- | --- | --- | --- | --- | --- |
| CpG1 | 3.0±0.6 | 2.3±1.0 | 8.341 (1, 59) | 0.005^**^ | 0.015^*^ |
| CpG2 | 3.4±0.6 | 2.6±0.7 | 20.047 (1, 59) | < 0.001^***^ | < 0.001^***^ |
| CpG3 | 5.3±0.7 | 5.2±0.5 | 0.315 (1, 59) | 0.577 | 0.649 |
| CpG4 | 3.5±0.8 | 3.0±0.6 | 9.668 (1, 59) | 0.003^**^ | 0.014^*^ |
| CpG5 | 4.4±1.2 | 4.7±0.7 | 0.336 (1, 59) | 0.564 | 0.649 |
| CpG6 | 3.5±1.8 | 4.8±1.3 | 2.435 (1, 59) | 0.124 | 0.223 |
| CpG7 | 3.1±1.4 | 4.0±1.1 | 4.245 (1, 59) | 0.044^*^ | 0.099 |
| CpG8 | 3.8±2.2 | 4.8±1.6 | 0.958 (1, 59) | 0.332 | 0.498 |
| Mean | 3.8±1.0 | 3.9±0.6 | 0.043 (1, 59) | 0.836 | 0.836 |

^a^Uncorrected p-value; ^b^False Discovery Rate adjusted p-value; ^c^Ranked ANCOVA: A statistic controlling for the potential confounding effects of education; OSSO, Other Specified Schizophrenia spectrum and Other psychotic disorder

* ＜ 0.05, ** ＜ 0.01, *** ＜ 0.001

**Table S7. Comparison of methylation rate of DRD2 between male and female in controls**

| **CpG sites** | **Male (n=10-13)** | **Female (n=31-34)** | **Mann-Whitney U** | **p-value^a^** | **p-value^b^** |
| --- | --- | --- | --- | --- | --- |
| CpG1 | 24.2±2.8 | 22.3±2.8 | 119.000 | 0.015^*^ | 0.225 |
| CpG2 | 32.9±3.9 | 31.4±2.4 | 158.000 | 0.134 | 0.613 |
| CpG3 | 9.9±1.1 | 9.6±1.5 | 190.000 | 0.461 | 0.693 |
| CpG4 | 11.7±1.6 | 11.0±1.6 | 119.000 | 0.286 | 0.613 |
| CpG5 | 6.1±1.3 | 5.8±1.3 | 173.000 | 0.254 | 0.613 |
| CpG6 | 5.4±0.7 | 5.3±1.2 | 197.500 | 0.576 | 0.693 |
| CpG7 | 4.5±0.8 | 4.6±0.7 | 245.000 | 0.568 | 0.693 |
| CpG8 | 4.5±0.6 | 4.4±0.8 | 191.000 | 0.475 | 0.693 |
| CpG9 | 11.0±1.8 | 10.7±1.6 | 199.000 | 0.601 | 0.693 |
| CpG10 | 15.7±2.1 | 16.0±2.1 | 236.000 | 0.721 | 0.748 |
| CpG11 | 15.5±1.5 | 15.4±1.8 | 207.500 | 0.748 | 0.748 |
| CpG12 | 17.4±1.3 | 17.7±1.3 | 254.000 | 0.433 | 0.693 |
| CpG13 | 11.4±1.4 | 11.1±1.4 | 170.000 | 0.225 | 0.613 |
| CpG14 | 5.1±0.8 | 4.8±0.6 | 169.500 | 0.221 | 0.613 |
| Mean | 12.5±1.1 | 12.2±0.9 | 164.000 | 0.175 | 0.613 |

^a^Uncorrected p-value

^b^False Discovery Rate adjusted p-value

* ＜ 0.05, ** ＜ 0.01, *** ＜ 0.001

**Table S8. Comparison of methylation rate of DRD2 between male and female in patients**

| **CpG sites** | **Male (n=21-20)** | **Female (n=28-30)** | **Mann-Whitney U** | **p-value^a^** | **p-value^b^** |
| --- | --- | --- | --- | --- | --- |
| CpG1 | 24.2±4.1 | 23.8±3.7 | 286.000 | 0.579 | 0.917 |
| CpG2 | 33.8±4.5 | 33.4±4.4 | 297.500 | 0.738 | 0.917 |
| CpG3 | 10.7±1.8 | 10.8±3.2 | 283.000 | 0.673 | 0.917 |
| CpG4 | 12.4±2.3 | 12.4±3.1 | 257.500 | 0.638 | 0.917 |
| CpG5 | 5.3±1.4 | 5.5±1.2 | 376.500 | 0.239 | 0.917 |
| CpG6 | 5.1±1.0 | 5.2±1.1 | 306.000 | 0.863 | 0.917 |
| CpG7 | 4.6±1.3 | 4.6±1.0 | 341.000 | 0.619 | 0.917 |
| CpG8 | 4.2±1.0 | 4.3±0.8 | 360.500 | 0.384 | 0.917 |
| CpG9 | 11.4±3.2 | 11.9±3.5 | 352.000 | 0.479 | 0.917 |
| CpG10 | 16.4±3.2 | 16.8±3.3 | 333.000 | 0.730 | 0.917 |
| CpG11 | 15.7±2.5 | 16.6±3.8 | 351.500 | 0.485 | 0.917 |
| CpG12 | 18.2±1.8 | 18.6±2.2 | 349.000 | 0.515 | 0.917 |
| CpG13 | 11.7±3.8 | 11.9±2.5 | 374.000 | 0.259 | 0.917 |
| CpG14 | 5.2±1.5 | 5.3±1.4 | 327.500 | 0.811 | 0.917 |
| Mean | 12.8±2.1 | 12.8±2.0 | 275.000 | 0.917 | 0.917 |

^a^Uncorrected p-value

^b^False Discovery Rate adjusted p-value

* ＜ 0.05, ** ＜ 0.01, *** ＜ 0.001

**Table S9. Comparison of methylation rate of NR3C1 between male and female in controls**

| **CpG sites** | **Male (n=13)** | **Female (n=34)** | **Mann-Whitney U** | **p-value^a^** | **p-value^b^** |
| --- | --- | --- | --- | --- | --- |
| CpG1 | 2.4±1.2 | 2.3±1.0 | 251.000 | 0.476 | 0.965 |
| CpG2 | 3.0±1.1 | 2.5±0.4 | 131.000 | 0.032^*^ | 0.288 |
| CpG3 | 5.3±0.6 | 5.2±0.5 | 219.500 | 0.972 | 0.972 |
| CpG4 | 3.2±1.1 | 2.9±0.3 | 169.500 | 0.221 | 0.965 |
| CpG5 | 4.8±0.8 | 4.6±0.7 | 217.500 | 0.934 | 0.972 |
| CpG6 | 4.8±1.4 | 4.7±1.2 | 188.500 | 0.439 | 0.965 |
| CpG7 | 4.1±1.0 | 3.9±1.1 | 240.500 | 0.643 | 0.965 |
| CpG8 | 5.0±1.7 | 4.7±1.6 | 216.500 | 0.915 | 0.972 |
| Mean | 4.1±0.8 | 3.9±0.5 | 196.000 | 0.552 | 0.965 |

^a^Uncorrected p-value

^b^False Discovery Rate adjusted p-value

* ＜ 0.05, ** ＜ 0.01, *** ＜ 0.001

**Table S10. Comparison of methylation rate of DRD2 between male and female in patients**

| **CpG sites** | **Male (n=21)** | **Female (n=29-30)** | **Mann-Whitney U** | **p-value^a^** | **p-value^b^** |
| --- | --- | --- | --- | --- | --- |
| CpG1 | 2.9±0.7 | 2.1±1.1 | 331.000 | 0.602 | 0.962 |
| CpG2 | 3.5±0.7 | 2.4±1.8 | 281.500 | 0.651 | 0.962 |
| CpG3 | 5.2±0.7 | 3.6±0.8 | 319.000 | 0.776 | 0.962 |
| CpG4 | 3.5±0.7 | 2.6±0.9 | 308.500 | 0.901 | 0.962 |
| CpG5 | 4.2±1.0 | 2.5±0.9 | 312.500 | 0.962 | 0.962 |
| CpG6 | 3.6±1.6 | 1.6±1.1 | 269.500 | 0.384 | 0.962 |
| CpG7 | 3.0±1.4 | 1.0±1.2 | 268.500 | 0.373 | 0.962 |
| CpG8 | 3.6±1.9 | 1.4±1.6 | 267.500 | 0.363 | 0.962 |
| Mean | 3.7±0.8 | 2.4±0.8 | 310.000 | 0.914 | 0.962 |

^a^Uncorrected p-value

^b^False Discovery Rate adjusted p-value

* ＜ 0.05, ** ＜ 0.01, *** ＜ 0.001

**Table S11. Correlation between methylation rate of DRD2 and clinical parameters in patients^a^**

| **CpG sites** | **BS-E (n=47)** | | **BS-C (n=47)** | | **BS-T (n=47)** | | **ETI-SF-G (n=46)** | | **ETI-SF-P (n=46)** | | **ETI-SF-E (n=46)** | | **ETI-SF-S (N=46)** | | **ETI-SF-T (N=46)** | | **PANSS-P (n=48)** | | **PANSS-N (n=48)** | | **PANSS-G (n=48)** | | **PANSS-T (n=48)** | | |
| --- | --- | --- | --- | --- | --- | --- | --- | --- | --- | --- | --- | --- | --- | --- | --- | --- | --- | --- | --- | --- | --- | --- | --- | --- | --- |
|  | **r** | **p-value** | **r** | **p-value** | **r** | **p-value** | **r** | **p-value** | **r** | **p-value** | **r** | **p-value** | **r** | **p-value** | **r** | **p-value** | **r** | **p-value** | **r** | **p-value** | **r** | **p-value** | **r** | **p-value** |  |
| CpG 1 | 0.273 | 0.073 | 0.251 | 0.100 | 0.232 | 0.130 | 0.146 | 0.349 | 0.049 | 0.756 | 0.324 | 0.034^*^ | 0.245 | 0.113 | 0.225 | 0.146 | 0.083 | 0.590 | -0.115 | 0.453 | -0.095 | 0.535 | -0.108 | 0.479 |  |
| CpG 2 | 0.267 | 0.080 | 0.362 | 0.016^*^ | 0.322 | 0.033^*^ | 0.193 | 0.214 | 0.092 | 0.559 | 0.383 | 0.011^*^ | 0.226 | 0.144 | 0.298 | 0.053 | 0.089 | 0.559 | -0.116 | 0.449 | -0.038 | 0.804 | -0.061 | 0.692 |  |
| CpG 3 | 0.061 | 0.696 | 0.327 | 0.030^*^ | 0.187 | 0.225 | 0.168 | 0.280 | 0.004 | 0.981 | 0.318 | 0.038^*^ | 0.105 | 0.503 | 0.180 | 0.249 | 0.031 | 0.840 | -0.084 | 0.585 | -0.172 | 0.258 | -0.144 | 0.346 |  |
| CpG 4 | 0.135 | 0.381 | 0.281 | 0.065 | 0.178 | 0.248 | 0.139 | 0.372 | -0.005 | 0.972 | 0.252 | 0.102 | 0.163 | 0.295 | 0.145 | 0.352 | -0.084 | 0.582 | -0.037 | 0.809 | -0.171 | 0.260 | -0.188 | 0.216 |  |
| CpG 5 | 0.275 | 0.071 | 0.328 | 0.030^*^ | 0.308 | 0.042^*^ | 0.088 | 0.574 | 0.109 | 0.488 | 0.175 | 0.263 | 0.246 | 0.112 | 0.177 | 0.256 | 0.337 | 0.024^*^ | -0.120 | 0.431 | 0.233 | 0.124 | 0.162 | 0.288 |  |
| CpG 6 | 0.217 | 0.157 | 0.197 | 0.199 | 0.223 | 0.145 | 0.094 | 0.548 | -0.141 | 0.368 | 0.023 | 0.884 | 0.112 | 0.474 | 0.042 | 0.790 | 0.212 | 0.163 | 0.089 | 0.560 | 0.053 | 0.731 | 0.142 | 0.351 |  |
| CpG 7 | 0.213 | 0.165 | 0.210 | 0.171 | 0.221 | 0.149 | 0.060 | 0.704 | -0.009 | 0.952 | 0.049 | 0.757 | 0.082 | 0.602 | 0.035 | 0.826 | 0.218 | 0.150 | 0.088 | 0.566 | 0.207 | 0.173 | 0.208 | 0.169 |  |
| CpG 8 | 0.151 | 0.328 | 0.061 | 0.695 | 0.106 | 0.495 | -0.054 | 0.733 | -0.127 | 0.417 | -0.119 | 0.448 | -0.132 | 0.400 | -0.140 | 0.372 | 0.041 | 0.789 | -0.014 | 0.927 | 0.033 | 0.831 | 0.011 | 0.942 |  |
| CpG 9 | 0.303 | 0.045^*^ | 0.369 | 0.014^*^ | 0.363 | 0.016^*^ | -0.187 | 0.230 | -0.149 | 0.341 | 0.239 | 0.123 | -0.022 | 0.887 | -0.033 | 0.836 | 0.017 | 0.913 | 0.174 | 0.253 | -0.061 | 0.691 | 0.043 | 0.777 |  |
| CpG 10 | 0.274 | 0.072 | 0.361 | 0.016^*^ | 0.364 | 0.015^*^ | -0.073 | 0.644 | -0.079 | 0.616 | 0.219 | 0.158 | -0.054 | 0.732 | 0.046 | 0.769 | 0.144 | 0.346 | 0.225 | 0.138 | -0.015 | 0.922 | 0.148 | 0.333 |  |
| CpG 11 | 0.223 | 0.146 | 0.340 | 0.024^*^ | 0.328 | 0.030^*^ | -0.071 | 0.652 | -0.097 | 0.538 | 0.282 | 0.067 | -0.103 | 0.513 | 0.074 | 0.638 | 0.055 | 0.720 | 0.168 | 0.271 | -0.096 | 0.529 | 0.045 | 0.767 |  |
| CpG 12 | 0.200 | 0.193 | 0.241 | 0.115 | 0.262 | 0.086 | -0.015 | 0.923 | 0.026 | 0.868 | 0.220 | 0.156 | -0.023 | 0.884 | 0.122 | 0.436 | -0.025 | 0.872 | 0.163 | 0.284 | -0.162 | 0.288 | -0.081 | 0.598 |  |
| CpG 13 | 0.146 | 0.345 | 0.279 | 0.067 | 0.243 | 0.113 | -0.034 | 0.828 | -0.151 | 0.334 | 0.170 | 0.275 | -0.102 | 0.513 | 0.005 | 0.975 | -0.015 | 0.924 | 0.195 | 0.199 | -0.074 | 0.627 | 0.032 | 0.835 |  |
| CpG 14 | 0.193 | 0.210 | 0.323 | 0.033^*^ | 0.279 | 0.066 | -0.098 | 0.530 | -0.097 | 0.538 | 0.309 | 0.044 | 0.082 | 0.603 | 0.060 | 0.703 | 0.155 | 0.309 | 0.142 | 0.353 | 0.092 | 0.549 | 0.127 | 0.407 |  |
| Mean | 0.353 | 0.019^*^ | 0.418 | 0.005^**^ | 0.402 | 0.007^**^ | 0.054 | 0.733 | 0.020 | 0.897 | 0.326 | 0.033^*^ | 0.065 | 0.680 | 0.183 | 0.240 | 0.103 | 0.500 | 0.056 | 0.714 | -0.053 | 0.729 | -0.021 | 0.889 |  |

Note: BS-E, -C and -T, Brooding Scale-Emotion, -Cognition and -Total; ETI-SF-G, -P, -E, -S and T, Early trauma inventory self report-short form-General, -Physical, -Emotion, -Sexual and -Total; PANSS-P, -N, -Gand -T, Positive and negative syndrome scale –Positive, -Negative, -General and -Total.

p-value, Uncorrected p-value. ^a^Spearman partial correlation analysis was performed with a covariate of age, gender, and CPZ.

* ＜ 0.05, ** ＜ 0.01, *** ＜ 0.001

**Table S12. Correlation between methylation rate of NR3C1 and clinical parameters in patients^a^**

| **CpG sites** | **BS-E (n=48)** | | **BS-C (n=48)** | | **BS-T (n=48)** | | **ETI-SF-G (n=48)** | | **ETI-SF-P (n=48)** | | **ETI-SF-E (n=48)** | | **ETI-SF-S (N=48)** | | **ETI-SF-T (N=48)** | | **PANSS-P (n=50)** | | **PANSS-N (n=50)** | | **PANSS-G (n=50)** | | **PANSS-T (n=50)** | |
| --- | --- | --- | --- | --- | --- | --- | --- | --- | --- | --- | --- | --- | --- | --- | --- | --- | --- | --- | --- | --- | --- | --- | --- | --- |
|  | **r** | **p-value** | **r** | **p-value** | **r** | **p-value** | **r** | **p-value** | **r** | **p-value** | **r** | **p-value** | **r** | **p-value** | **r** | **p-value** | **r** | **p-value** | **r** | **p-value** | **r** | **p-value** | **r** | **p-value** |
| CpG 1 | -0.033 | 0.827 | -0.075 | 0.627 | -0.059 | 0.700 | -0.051 | 0.737 | 0.071 | 0.645 | -0.070 | 0.646 | -0.124 | 0.417 | -0.061 | 0.688 | 0.026 | 0.862 | -0.056 | 0.707 | -0.072 | 0.632 | -0.042 | 0.780 |
| CpG 2 | -0.137 | 0.369 | -0.132 | 0.389 | -0.147 | 0.334 | 0.115 | 0.452 | -0.087 | 0.572 | -0.223 | 0.141 | -0.161 | 0.291 | -0.134 | 0.381 | 0.112 | 0.455 | -0.129 | 0.389 | -0.108 | 0.469 | -0.018 | 0.902 |
| CpG 3 | 0.022 | 0.885 | -0.137 | 0.368 | -0.037 | 0.809 | 0.069 | 0.655 | 0.063 | 0.680 | 0.044 | 0.773 | 0.127 | 0.407 | 0.123 | 0.421 | 0.093 | 0.533 | -0.017 | 0.911 | -0.051 | 0.733 | -0.031 | 0.838 |
| CpG 4 | -0.018 | 0.907 | -0.010 | 0.948 | -0.013 | 0.932 | 0.197 | 0.194 | 0.093 | 0.542 | 0.014 | 0.928 | -0.011 | 0.942 | 0.092 | 0.547 | 0.045 | 0.765 | 0.041 | 0.786 | -0.102 | 0.495 | 0.016 | 0.917 |
| CpG 5 | 0.167 | 0.272 | 0.217 | 0.152 | 0.247 | 0.102 | -0.175 | 0.249 | 0.199 | 0.190 | 0.149 | 0.328 | 0.171 | 0.262 | 0.067 | 0.663 | -0.213 | 0.150 | 0.416 | 0.004^**^ | -0.021 | 0.888 | 0.084 | 0.574 |
| CpG 6 | 0.119 | 0.434 | 0.252 | 0.094 | 0.217 | 0.152 | -0.169 | 0.267 | 0.180 | 0.236 | 0.126 | 0.411 | 0.133 | 0.382 | 0.051 | 0.739 | -0.198 | 0.183 | 0.301 | 0.040^*^ | -0.017 | 0.912 | 0.042 | 0.780 |
| CpG 7 | 0.172 | 0.257 | 0.242 | 0.109 | 0.240 | 0.112 | 0.010 | 0.948 | 0.262 | 0.082 | 0.168 | 0.269 | 0.091 | 0.551 | 0.140 | 0.360 | -0.172 | 0.249 | 0.309 | 0.035^*^ | 0.096 | 0.523 | 0.092 | 0.538 |
| CpG 8 | 0.164 | 0.283 | 0.207 | 0.172 | 0.212 | 0.162 | -0.069 | 0.653 | 0.210 | 0.166 | 0.159 | 0.298 | 0.044 | 0.772 | 0.093 | 0.544 | -0.152 | 0.309 | 0.352 | 0.015^*^ | 0.012 | 0.934 | 0.088 | 0.557 |
| Mean | 0.059 | 0.702 | 0.106 | 0.488 | 0.104 | 0.495 | -0.072 | 0.639 | 0.157 | 0.304 | 0.098 | 0.521 | 0.092 | 0.549 | 0.040 | 0.792 | -0.084 | 0.575 | 0.236 | 0.111 | 0.043 | 0.772 | 0.088 | 0.557 |

Note: BS-E, -C and -T, Brooding Scale-Emotion, -Cognition and -Total; ETI-SF-G, -P, -E, -S and T, Early trauma inventory self report-short form-General, -Physical, -Emotion, -Sexual and -Total; PANSS-P, -N, -G and -T, Positive and negative syndrome scale –Positive, -Negative, -General and -Total. p-value, Uncorrected p-value ^b^ Spearman partial correlation analysis was performed with a covariate of age, gender.

* ＜ 0.05, ** ＜ 0.01, *** ＜ 0.001

**Table S13. Correlation between methylation rate of DRD2 and clinical parameters in between antipsychotic-naïve and -free patients^b^**

| **CpG sites** | **BS-E (n=30)** | | **BS-C (n=30)** | | **BS-T (n=30)** | | **ETI-SF-G (n=29)** | | **ETI-SF-P (n=29)** | | **ETI-SF-E (n=29)** | | **ETI-SF-S (N=29)** | | **ETI-SF-T (N=29)** | | **PANSS-P (n=31)** | | **PANSS-N (n=31)** | | **PANSS-G (n=31)** | | **PANSS-T (n=31)** | |
| --- | --- | --- | --- | --- | --- | --- | --- | --- | --- | --- | --- | --- | --- | --- | --- | --- | --- | --- | --- | --- | --- | --- | --- | --- |
|  | **r** | **p-value^a^** | **r** | **p-value^a^** | **r** | **p-value^a^** | **r** | **p-value^a^** | **r** | **p-value^a^** | **r** | **p-value^a^** | **r** | **p-value^a^** | **r** | **p-value^a^** | **r** | **p-value^a^** | **r** | **p-value^a^** | **r** | **p-value^a^** | **r** | **p-value^a^** |
| CpG 1 | 0.419^*^ | 0.132 | 0.368 | 0.081 | 0.362 | 0.081 | 0.296 | 0.425 | 0.249 | 0.792 | 0.474^*^ | 0.042^*^ | 0.339 | 0.420 | 0.455^*^ | 0.065 | 0.064 | 0.925 | -0.108 | 0.736 | -0.205 | 0.924 | -0.120 | 0.669 |
| CpG 2 | 0.292 | 0.198 | 0.446^*^ | 0.037^*^ | 0.368 | 0.081 | 0.390^*^ | 0.425 | 0.182 | 0.907 | 0.437^*^ | 0.042^*^ | 0.188 | 0.668 | 0.481^*^ | 0.065 | 0.083 | 0.911 | -0.226 | 0.512 | -0.184 | 0.924 | -0.141 | 0.634 |
| CpG 3 | 0.251 | 0.229 | 0.414^*^ | 0.048^*^ | 0.341 | 0.095 | 0.290 | 0.425 | 0.321 | 0.792 | 0.441^*^ | 0.042^*^ | 0.388^*^ | 0.340 | 0.454^*^ | 0.065 | 0.225 | 0.787 | -0.105 | 0.736 | -0.164 | 0.924 | -0.067 | 0.748 |
| CpG 4 | 0.262 | 0.223 | 0.320 | 0.122 | 0.253 | 0.208 | 0.314 | 0.425 | 0.196 | 0.907 | 0.416^*^ | 0.052 | 0.410^*^ | 0.340 | 0.378 | 0.152 | -0.048 | 0.928 | -0.181 | 0.649 | -0.370^*^ | 0.720 | -0.291 | 0.397 |
| CpG 5 | 0.341 | 0.190 | 0.322 | 0.122 | 0.360 | 0.081 | 0.133 | 0.858 | 0.251 | 0.792 | 0.107 | 0.639 | 0.155 | 0.668 | 0.189 | 0.398 | 0.360 | 0.274 | -0.045 | 0.874 | 0.301 | 0.841 | 0.244 | 0.433 |
| CpG 6 | 0.462^*^ | 0.100 | 0.307 | 0.129 | 0.412^*^ | 0.063 | 0.298 | 0.425 | -0.019 | 0.923 | 0.163 | 0.522 | 0.037 | 0.914 | 0.282 | 0.231 | 0.416^*^ | 0.274 | 0.159 | 0.677 | 0.077 | 0.924 | 0.286 | 0.397 |
| CpG 7 | 0.297 | 0.198 | 0.237 | 0.241 | 0.297 | 0.144 | 0.131 | 0.858 | 0.136 | 0.923 | 0.083 | 0.681 | -0.170 | 0.668 | 0.142 | 0.514 | 0.368^*^ | 0.274 | 0.050 | 0.874 | 0.244 | 0.924 | 0.261 | 0.427 |
| CpG 8 | 0.193 | 0.324 | 0.149 | 0.450 | 0.171 | 0.384 | -0.006 | 0.977 | -0.021 | 0.923 | -0.138 | 0.569 | -0.287 | 0.550 | -0.097 | 0.630 | 0.215 | 0.787 | -0.007 | 0.970 | 0.098 | 0.924 | 0.158 | 0.634 |
| CpG 9 | 0.387^*^ | 0.152 | 0.524^**^ | 0.032^*^ | 0.497^**^ | 0.035^*^ | -0.094 | 0.858 | 0.086 | 0.923 | 0.507^**^ | 0.035^*^ | 0.130 | 0.668 | 0.210 | 0.368 | 0.020 | 0.964 | 0.342 | 0.261 | -0.051 | 0.924 | 0.151 | 0.634 |
| CpG 10 | 0.373 | 0.152 | 0.474^*^ | 0.033^*^ | 0.507^**^ | 0.035^*^ | 0.030 | 0.945 | 0.064 | 0.923 | 0.445^*^ | 0.042^*^ | 0.051 | 0.914 | 0.257 | 0.267 | 0.192 | 0.788 | 0.402^*^ | 0.180 | 0.034 | 0.924 | 0.332 | 0.397 |
| CpG 11 | 0.320 | 0.197 | 0.463^*^ | 0.033^*^ | 0.458^*^ | 0.053 | 0.073 | 0.858 | 0.050 | 0.923 | 0.466^*^ | 0.042^*^ | 0.008 | 0.968 | 0.296 | 0.231 | 0.174 | 0.788 | 0.391^*^ | 0.180 | 0.040 | 0.924 | 0.298 | 0.397 |
| CpG 12 | 0.241 | 0.233 | 0.467^*^ | 0.033^*^ | 0.383^*^ | 0.081 | 0.118 | 0.858 | 0.161 | 0.907 | 0.376 | 0.073 | 0.151 | 0.668 | 0.366 | 0.152 | 0.009 | 0.964 | 0.291 | 0.366 | -0.117 | 0.924 | 0.062 | 0.748 |
| CpG 13 | 0.313 | 0.197 | 0.415^*^ | 0.048^*^ | 0.424^*^ | 0.061 | 0.110 | 0.858 | 0.060 | 0.923 | 0.408^*^ | 0.052 | 0.125 | 0.668 | 0.290 | 0.231 | 0.107 | 0.869 | 0.440^*^ | 0.180 | 0.010 | 0.960 | 0.314 | 0.397 |
| CpG 14 | 0.281 | 0.202 | 0.499^**^ | 0.033^*^ | 0.435^*^ | 0.061 | 0.066 | 0.858 | 0.119 | 0.923 | 0.535^**^ | 0.031^*^ | 0.199 | 0.668 | 0.310 | 0.231 | 0.108 | 0.869 | 0.277 | 0.366 | 0.049 | 0.924 | 0.161 | 0.634 |
| Mean | 0.501^**^ | 0.099 | 0.544^**^ | 0.032^*^ | 0.546^**^ | 0.035^*^ | 0.268 | 0.441 | 0.306 | 0.792 | 0.535^**^ | 0.031^*^ | 0.217 | 0.668 | 0.510^**^ | 0.065 | 0.141 | 0.869 | 0.146 | 0.677 | -0.074 | 0.924 | 0.063 | 0.748 |

Note: BS-E, -C and -T, Brooding Scale-Emotion, -Cognition and -Total; ETISF-G, -P, -E, -S and T, Early trauma inventory self report-short form-General, -Physical, -Emotion, -Sexual and -Total; PANSS-P, -N, -G and -T, Positive and negative syndrome scale –Positive, -Negative, -General and -Total. ^a^False Discovery Rate adjusted p-value. ^b^ Spearman partial correlation analysis was performed with a covariate of age, gender.

* ＜ 0.05, ** ＜ 0.01, *** ＜ 0.001

| **CpG sites** | **BS-E (n=41)** | | **BS-C (n=41)** | | **BS-T (n=41)** | | **ETI-SF-G (n=41)** | | **ETI-SF-P (n=41)** | | **ETI-SF-E (n=41)** | | **ETI-SF-S (N=41)** | | **ETI-SF-T (N=41)** | |
| --- | --- | --- | --- | --- | --- | --- | --- | --- | --- | --- | --- | --- | --- | --- | --- | --- |
|  | **r** | **p-value** | **r** | **p-value** | **r** | **p-value** | **r** | **p-value** | **r** | **p-value** | **r** | **p-value** | **r** | **p-value** | **r** | **p-value** |
| CpG 1 | 0.208 | 0.205 | 0.218 | 0.183 | 0.239 | 0.144 | 0.072 | 0.662 | 0.263 | 0.105 | 0.184 | 0.263 | 0.092 | 0.576 | 0.186 | 0.257 |
| CpG 2 | -0.032 | 0.845 | -0.175 | 0.288 | -0.088 | 0.593 | 0.125 | 0.449 | 0.086 | 0.602 | -0.116 | 0.482 | 0.247 | 0.129 | 0.087 | 0.597 |
| CpG 3 | 0.391 | 0.014^*^ | 0.169 | 0.305 | 0.333 | 0.038^*^ | 0.375 | 0.019^*^ | 0.350 | 0.029^*^ | 0.382 | 0.016^*^ | 0.071 | 0.667 | 0.441 | 0.005^**^ |
| CpG 4 | 0.068 | 0.682 | 0.043 | 0.795 | 0.065 | 0.694 | 0.223 | 0.173 | 0.369 | 0.021^*^ | 0.144 | 0.383 | 0.232 | 0.155 | 0.358 | 0.025^*^ |
| CpG 5 | 0.172 | 0.296 | -0.106 | 0.521 | 0.058 | 0.728 | 0.181 | 0.271 | 0.190 | 0.246 | 0.109 | 0.510 | 0.234 | 0.151 | 0.260 | 0.110 |
| CpG 6 | 0.093 | 0.575 | 0.113 | 0.492 | 0.118 | 0.476 | 0.199 | 0.224 | 0.145 | 0.379 | 0.106 | 0.519 | 0.114 | 0.489 | 0.194 | 0.237 |
| CpG 7 | -0.160 | 0.331 | -0.148 | 0.368 | -0.197 | 0.230 | 0.147 | 0.373 | 0.223 | 0.173 | 0.023 | 0.889 | -0.106 | 0.519 | 0.149 | 0.367 |
| CpG 8 | -0.055 | 0.739 | 0.028 | 0.864 | -0.006 | 0.972 | 0.243 | 0.136 | 0.218 | 0.182 | 0.135 | 0.414 | 0.180 | 0.273 | 0.217 | 0.184 |
| CpG 9 | -0.250 | 0.125 | -0.218 | 0.182 | -0.271 | 0.095 | -0.117 | 0.479 | -0.045 | 0.785 | -0.122 | 0.458 | 0.021 | 0.898 | -0.129 | 0.434 |
| CpG 10 | -0.323 | 0.045^*^ | -0.356 | 0.026^*^ | -0.377 | 0.018^*^ | -0.049 | 0.767 | 0.074 | 0.654 | -0.155 | 0.345 | -0.130 | 0.430 | -0.080 | 0.629 |
| CpG 11 | -0.295 | 0.068 | -0.432 | 0.006^**^ | -0.367 | 0.022^*^ | -0.181 | 0.269 | 0.071 | 0.669 | -0.303 | 0.061 | 0.044 | 0.790 | -0.117 | 0.477 |
| CpG 12 | -0.243 | 0.136 | -0.325 | 0.043^*^ | -0.335 | 0.037^*^ | 0.042 | 0.800 | 0.075 | 0.650 | -0.115 | 0.484 | -0.255 | 0.117 | 0.013 | 0.936 |
| CpG 13 | -0.213 | 0.193 | -0.450 | 0.004^**^ | -0.351 | 0.028^*^ | 0.036 | 0.826 | 0.325 | 0.043^*^ | -0.013 | 0.940 | -0.056 | 0.736 | 0.197 | 0.229 |
| CpG 14 | -0.220 | 0.178 | -0.303 | 0.061 | -0.299 | 0.064 | -0.140 | 0.396 | 0.161 | 0.327 | 0.020 | 0.905 | -0.105 | 0.524 | -0.017 | 0.919 |
| Mean | -0.026 | 0.877 | -0.144 | 0.382 | -0.074 | 0.653 | 0.096 | 0.562 | 0.260 | 0.110 | 0.066 | 0.688 | 0.170 | 0.301 | 0.197 | 0.229 |

**Table S14. Correlation between methylation rate of DRD2 and clinical parameters in controls^a^**

Note: BS-E, -C and -T, Brooding Scale-Emotion, -Cognition and -Total; ETI-SF-G, -P, -E, -S and T, Early trauma inventory self report-short form-General, -Physical, -Emotion, -Sexual and -Total.

p-value, Uncorrected p-value. ^a^Spearman partial correlation analysis was performed with a covariate of age and gender.

* ＜ 0.05, ** ＜ 0.01, *** ＜ 0.001

**Table S15. Correlation between methylation rate of NR3C1 and clinical parameters in controls^a^**

| **CpG sites** | **BS-E (n=47)** | | **BS-C (n=47)** | | **BS-T (n=47)** | | **ETI-SF-G (n=47)** | | **ETI-SF-P (n=47)** | | **ETI-SF-E (n=47)** | | **ETI-SF-S (N=47)** | | **ETI-SF-T (N=47)** | | |
| --- | --- | --- | --- | --- | --- | --- | --- | --- | --- | --- | --- | --- | --- | --- | --- | --- | --- |
|  | **r** | **p-value** | **r** | **p-value** | **r** | **p-value** | **r** | **p-value** | **r** | **p-value** | **r** | **p-value** | **r** | **p-value** | **r** | **p-value** |  |
| CpG 1 | 0.262 | 0.083 | -0.278 | 0.064 | 0.043 | 0.778 | 0.017 | 0.913 | -0.033 | 0.830 | 0.135 | 0.377 | 0.011 | 0.945 | 0.057 | 0.710 |  |
| CpG 2 | 0.077 | 0.613 | -0.249 | 0.100 | -0.075 | 0.626 | -0.018 | 0.906 | -0.116 | 0.448 | -0.079 | 0.604 | -0.250 | 0.097 | -0.146 | 0.338 |  |
| CpG 3 | 0.248 | 0.101 | 0.059 | 0.699 | 0.177 | 0.245 | 0.090 | 0.555 | -0.173 | 0.256 | 0.143 | 0.350 | -0.064 | 0.675 | -0.056 | 0.716 |  |
| CpG 4 | -0.028 | 0.857 | -0.310 | 0.038 | -0.199 | 0.190 | -0.070 | 0.647 | -0.030 | 0.843 | 0.103 | 0.499 | -0.379 | 0.010 | -0.113 | 0.460 |  |
| CpG 5 | 0.045 | 0.768 | 0.169 | 0.267 | 0.132 | 0.386 | 0.106 | 0.488 | 0.072 | 0.640 | 0.166 | 0.275 | -0.031 | 0.841 | 0.081 | 0.595 |  |
| CpG 6 | 0.048 | 0.752 | 0.012 | 0.937 | 0.068 | 0.655 | 0.071 | 0.643 | 0.038 | 0.806 | 0.021 | 0.890 | -0.125 | 0.414 | 0.035 | 0.820 |  |
| CpG 7 | 0.175 | 0.250 | 0.264 | 0.079 | 0.243 | 0.108 | 0.067 | 0.664 | 0.060 | 0.697 | 0.077 | 0.616 | -0.036 | 0.815 | 0.063 | 0.682 |  |
| CpG 8 | 0.162 | 0.288 | 0.106 | 0.489 | 0.151 | 0.323 | 0.142 | 0.351 | -0.044 | 0.775 | 0.031 | 0.840 | 0.008 | 0.957 | 0.094 | 0.539 |  |
| Mean | 0.195 | 0.199 | 0.067 | 0.662 | 0.166 | 0.275 | 0.063 | 0.683 | -0.044 | 0.772 | 0.063 | 0.681 | -0.089 | 0.560 | 0.002 | 0.991 |  |

Note: BS-E, -C and -T, Brooding Scale-Emotion, -Cognition and -Total; ETI-SF-G, -P, -E, -S and T, Early trauma inventory self report-short form-General, -Physical, -Emotion, -Sexual and -Total.

p-value, Uncorrected p-value. ^a^Spearman partial correlation analysis was performed with a covariate of age, gender.

* ＜ 0.05, ** ＜ 0.01, *** ＜ 0.001

| **CpG sites** | **Age (n=48)** | | |
| --- | --- | --- | --- |
|  | **r** | **p-value^a^** | **p-value^b^** |
| CpG 1 | 0.512 | < 0.001^***^ | 0.004 |
| CpG 2 | 0.351 | 0.017 | 0.027 |
| CpG 3 | 0.467 | 0.001 | 0.004 |
| CpG 4 | 0.142 | 0.347 | 0.347 |
| CpG 5 | 0.433 | 0.003 | 0.006 |
| CpG 6 | 0.277 | 0.063 | 0.072 |
| CpG 7 | 0.348 | 0.018 | 0.027 |
| CpG 8 | 0.217 | 0.148 | 0.159 |
| CpG 9 | 0.424 | 0.003 | 0.006 |
| CpG 10 | 0.491 | 0.001 | 0.004 |
| CpG 11 | 0.447 | 0.002 | 0.005 |
| CpG 12 | 0.313 | 0.034 | 0.046 |
| CpG 13 | 0.461 | 0.001 | 0.004 |
| CpG 14 | 0.292 | 0.049 | 0.061 |
| Mean | 0.482 | 0.001 | 0.004 |

**Table S16.** **Correlation between methylation rate of DRD2 and age in patients^c^**

^a^Uncorrected p-value

^b^False Discovery Rate adjusted p-value

^c^Spearman partial correlation analysis was performed with a covariate of gender and CPZ.

* ＜ 0.05, ** ＜ 0.01, *** ＜ 0.001

**Table S17.** **Correlation between methylation rate of DRD2 and age in controls^c^**

| **CpG sites** | **Age (n=41)** | | |
| --- | --- | --- | --- |
|  | **r** | **p-value^a^** | **p-value^b^** |
| CpG 1 | 0.130 | 0.426 | 0.456 |
| CpG 2 | 0.250 | 0.120 | 0.151 |
| CpG 3 | 0.255 | 0.112 | 0.151 |
| CpG 4 | 0.090 | 0.583 | 0.583 |
| CpG 5 | 0.205 | 0.205 | 0.237 |
| CpG 6 | 0.308 | 0.053 | 0.089 |
| CpG 7 | 0.340 | 0.032 | 0.069 |
| CpG 8 | 0.330 | 0.038 | 0.070 |
| CpG 9 | 0.554 | < 0.001^***^ | 0.001 |
| CpG 10 | 0.547 | < 0.001^***^ | 0.001 |
| CpG 11 | 0.559 | < 0.001^***^ | 0.001 |
| CpG 12 | 0.293 | 0.067 | 0.100 |
| CpG 13 | 0.551 | < 0.001^***^ | 0.001 |
| CpG 14 | 0.477 | 0.002 | 0.005 |
| Mean | 0.490 | 0.001 | 0.004 |

^a^Uncorrected p-value

^b^False Discovery Rate adjusted p-value

^c^Spearman partial correlation analysis was performed with a covariate of gender.

* ＜ 0.05, ** ＜ 0.01, *** ＜ 0.001

**Table S18. Correlation between methylation rate of NR3C1 and age in patients^c^**

| **CpG sites** | **Age (n=50)** | | |
| --- | --- | --- | --- |
|  | **r** | **p-value^a^** | **p-value^b^** |
| CpG 1 | -0.123 | 0.404 | 0.520 |
| CpG 2 | -0.202 | 0.170 | 0.520 |
| CpG 3 | -0.090 | 0.544 | 0.612 |
| CpG 4 | -0.162 | 0.272 | 0.520 |
| CpG 5 | -0.134 | 0.365 | 0.520 |
| CpG 6 | -0.208 | 0.156 | 0.520 |
| CpG 7 | -0.009 | 0.951 | 0.951 |
| CpG 8 | -0.133 | 0.369 | 0.520 |
| Mean | -0.177 | 0.228 | 0.520 |

^a^Uncorrected p-value

^b^False Discovery Rate adjusted p-value

^c^Spearman partial correlation analysis was performed with a covariate of gender and CPZ.

* ＜ 0.05, ** ＜ 0.01, *** ＜ 0.001

| **CpG sites** | **Age (n=47)** | | |
| --- | --- | --- | --- |
|  | **r** | **p-value^a^** | **p-value^b^** |
| CpG 1 | 0.199 | 0.184 | 0.661 |
| CpG 2 | 0.142 | 0.347 | 0.661 |
| CpG 3 | -0.136 | 0.367 | 0.661 |
| CpG 4 | -0.171 | 0.255 | 0.661 |
| CpG 5 | -0.195 | 0.193 | 0.661 |
| CpG 6 | -0.041 | 0.784 | 0.891 |
| CpG 7 | -0.040 | 0.792 | 0.891 |
| CpG 8 | 0.056 | 0.711 | 0.891 |
| Mean | -0.002 | 0.989 | 0.989 |

**Table 19. Correlation between methylation rate of NR3C1 and age in controls^c^**

^a^Uncorrected p-value

^b^False Discovery Rate adjusted p-value

^c^Spearman partial correlation analysis was performed with a covariate of gender.

* ＜ 0.05, ** ＜ 0.01, *** ＜ 0.001
